# Characterization of IncC Plasmids in Enterobacterales of Food-Producing Animals Originating From China

**DOI:** 10.3389/fmicb.2020.580960

**Published:** 2020-10-27

**Authors:** Yu Zhang, Chang-Wei Lei, Xuan Chen, Tian-Ge Yao, Jing-Wen Yu, Wan-Long Hu, Xuan Mao, Hong-Ning Wang

**Affiliations:** College of Life Sciences, Key Laboratory of Bio-Resource and Eco-Environment of Ministry of Education, Animal Disease Prevention and Food Safety Key Laboratory of Sichuan Province, Sichuan University, Chengdu, China

**Keywords:** antibiotic resistance, food-producing animal, insertion sequence, inversion, IncC plasmids

## Abstract

Incompatibility group C (IncC) plasmids have received attention due to their broad host range and because they harbor key antibiotic resistance genes. Because these resistance genes can spread from food-producing animals to human, the proliferation of these plasmids represents a public health risk. In this study, a total of 20 IncC plasmids were collected from food-producing animals in China, and characterized by Oxford Nanopore Technologies long-read sequencing. Based on four key differences of the IncC backbone, 4 IncC plasmids were classified as type 1, 15 were classified as type 1/2 hybrid, and one was classified as type 2. The 15 type 1/2 hybrids were further divided into 13 type 1/2a and 2 type 1/2b, based on sequence differences arising from different homologous recombination events between type 1 and type 2 IncC backbones. Genome comparison of accessory resistance modules showed that different IncC plasmids exhibited various phenotypes via loss and gain of diverse modules, mainly within the *bla*_*CMY*_-carrying region, and two antibiotic resistance islands designated ARI-A and ARI-B. Interestingly, in addition to insertion and deletion events, IS*26* or IS*1294*-mediated large sequence inversions were found in the IncC genome of the 4 type1/2a plasmids, suggesting that insertion sequence-mediated rearrangements also promote the diversity of the IncC genome. This study provides insight into the structural diversification and multidrug resistance of IncC plasmids identified from food-producing animals in China.

## Introduction

The emergence and spread of antimicrobial resistance in bacteria pose a serious threat to global health and food security (Laxminarayan et al., 2016; Liu et al., 2016; He et al., 2019). Consequently, the WHO officially recognized antimicrobial resistance as a significant threat to global health in 2019.^1^ Mobile genetic elements (MGEs) are responsible for the capture, accumulation, and dissemination of resistance genes. Plasmids are important vehicles for other MGEs and acquired antimicrobial resistance genes associated with these elements in both Gram-negative and Gram-positive genera of bacteria (Partridge et al., 2018). Accordingly, plasmids play a significant role in the worldwide dissemination of multidrug resistance (MDR; Carattoli, 2009; Chen et al., 2018; Partridge et al., 2018; Pinilla-Redondo et al., 2018) and the evolution of antimicrobial resistance in bacteria (San Millan et al., 2016; San Millan, 2018). Incompatibility group C (IncC) plasmids were first reported in the 1960s, and were grouped with the related IncA plasmid RA1 as the A–C complex in 1974, with the term “IncA/C” subsequently coined in the late 1980s (Datta and Hedges, 1973; Hedges, 1974; Couturier et al., 1988; Harmer and Hall, 2015). More recently, the original names of IncA and IncC are becoming more widely used to more accurately describe compatibility and backbone divergence (Harmer and Hall, 2015; Ambrose et al., 2018a, b). IncC plasmids occur widely in Gram-negative bacteria that are resistant to multiple antibiotics, indicating a broad host range (Partridge et al., 2018). These plasmids have received most attention due to their association with the dissemination of the *bla*_*CMY*_ cephalosporinase and *bla*_*NDM*_ carbapenemase genes (Mulvey et al., 2009; Mataseje et al., 2010; Guo et al., 2014; Harmer and Hall, 2014), and these plasmids can harbor genes conferring resistance to several different antibiotics, such as aminoglycoside and fluoroquinolone resistance genes, allowing resistance to many clinically useful aminoglycosides and fluoroquinolone (Shoma et al., 2014; Wasyl et al., 2015).

An approximately 1% nucleotide divergence of the IncC plasmids backbones separate a group including type 1, and a group including type2, which are represented by reference plasmids pR148 (Del Castillo et al., 2013), and pR55 (Doublet et al., 2012), respectively. These two lineages are also distinguished by two variable regions (R1 and R2), *orf1832* in type 1 or *orf1847* in type 2, *rhs1* in type 1 or *rhs2* in type 2, and two additional sequences (i1 and i2) in the type 2 IncC backbone (Harmer and Hall, 2014). Type 1 can be further divided into two sub-groups, type 1a and 1b, based on the presence (1a) or absence (1b) of a diverged segment that contains Single-nucleotide polymorphisms (SNPs) concentrated in a 14.5 kb part of the IncC genome (Harmer and Hall, 2017). More recently, a small number of novel type 1/2 hybrid IncC plasmids were reported (Harmer and Hall, 2015; Lei et al., 2017; Papagiannitsis et al., 2017). These plasmids share backbone features with both type 1 and type 2 plasmids, indicating that despite strong entry exclusion and incompatibility, homologous recombination can occur between type 1 and type 2 IncC plasmids (Ambrose et al., 2018b). One study found that IS*26* can act to generate an the IncC-IncX3-cointegrated plasmid (Li et al., 2019), with expanded resistance profile, effectively broadening the host spectrum of the resistance-encoding mobile elements.

For a long time, due to the importance of *bla*_*CMY–*__2_, *bla*_*NDM*_, or other carbapenemase resistance genes, the reports and sequencing had a clear preference for type1a IncC plasmids (Harmer and Hall, 2015; Ambrose et al., 2018b). Ever-increasing studies of IncC plasmids have provided a fascinating insight into their evolutionary history (Ambrose et al., 2018b; Cheng et al., 2019), but the reports about animal derived IncC plasmids remain sporadic and limited. To further characterize plasmid strategies to disseminate antibiotic genes, we characterized 20 IncC plasmids identified from food-producing animals in China. Our sequencing results allow a comprehensive genomic comparison, providing insight into the structural diversification and MDR capacity of IncC plasmids found from food-producing animals in China.

## Materials and Methods

### Bacterial Strains and the detection of IncC plasmids

A total of non-duplicate 870 Enterobacterales strains (369 *Escherichia coli* strains, 212 *Klebsiella pneumoniae* strains, 125 *Salmonella enterica* strains, 56 *Proteus mirabilis* strains, 43 *Proteus vulgaris* strains, 38 *Citrobacter* strains, and 27 *Enterobacter cloacae* strains) were used for IncC plasmid identification in this study. The strains were recovered from samples of feces, diseased tissues, or cloacal or anal swabs of animals from 58 livestock farms (27 poultry and 31 swine farms) located in 16 provinces in China, with samples collected between 2015 and 2019 (Supplementary Table S1). All isolates were identified by BD Phoenix 100 diagnostic systems (Sparks, MD, United States). IncC plasmids were identified by PCR amplification with primer pair C-F (5′–3′ GAGAACCAAAGACAAAGACCTGGA)/C-R (5′–3′ ACGACAAACCTGAATTGCCTCCTT) that targets *repA* gene (Carattoli et al., 2005). And amplification was carried out with the following thermal cycling conditions: 3 min at 95°C and 35 cycles of amplification consisting of 25 s at 94°C, 25 s at 55°C, and 10 s at 72°C, with 5 min at 72°C for the final extension. The positive isolates identified by PCR were sequenced by whole genome sequencing (WGS, see Section “DNA Extraction, Purification and Library Preparation”) combined PlasmidFinder 2.1 analysis (Carattoli et al., 2014) to further determine the presence of the IncC plasmid.

### Antimicrobial Susceptibility Testing

Bacterial antimicrobial susceptibility was determined by the broth dilution or agar dilution method (for fosfomycin, using agar media supplemented with 25 μg/mL of glucose-6-phosphate) according to CLSI (Clsi, 2018b) and Veterinary CLSI (Clsi, 2018a) guidelines. The tested antimicrobial agents included ampicillin (AMP), cefoxitin (FOX), cefotaxime (CTX), ceftriaxone (CRO), imipenem (IPM), amoxicillin-clavulanic acid (AMC), florfenicol (FFC), ciprofloxacin (CIP), gentamicin (GEN), doxycycline (DOX), sulfamethoxazole (SUL), trimethoprim (TMP), fosfomycin (FOS), and polymyxin B (POL). *E. coli* ATCC25922 was used as a quality control strain.

### DNA Extraction, Purification and Library Preparation

Genomic DNAs of IncC-positive strains were extracted by using a MiniBEST Bacteria Genomic DNA Extraction Kit (TaKaRa, Dalian, China), and automatically recovered using the BluePippin (Sage science, United States). The quality and quantity of genomic DNA were confirmed using a Qubit 2.0 fluorometer (Life Technologies). PromethION and Illumina sequencing library preparation were performed using SQK-LSK109 ligation genomic DNA kit (Oxford Nanopore Technologies, United Kingdom) and NEBNext^®^Ultra^TM^ DNA Library Prep Kitfor Illumina (NEB, United States), respectively.

### Whole Genome Sequencing, Assembly, MLST, and SNP Analysis

Whole genome sequencing was performed using the Illumina PE150 platform (350-bp paired-end reads) combined with the Nanopore PromethION 48 platform (Novogene, China), and the read length and depth for each sample was shown in Supplementary Table S2. The quality check for sequencing reads were performed by NanoPlot 1.3.1 soft (Q > 7). The reads were assembled using the software SPAdes_3.12.0 and Unicycler GPLv3 (Wick et al., 2017),^2^ and the complete nucleotide sequences of all IncC plasmids were verified by PCR. The assembled sequences were analyzed to identify multi-locus sequence typing (MLST) by the MLST 2.0 (Larsen et al., 2012).^3^ Single-nucleotide polymorphism alignments were computed using the CSI phylogeny 1.4 pipeline (Kaas et al., 2014) according to the default parameters.^4^

### Sequence Annotation and Comparison

The complete plasmid sequences were annotated with the Rapid Annotation using Subsystem Technology (RAST) tool (Brettin et al., 2015) and the NCBI BLAST algorithm.^5^ For analysis and annotation of resistance genes, mobile elements, and other features, BLAST, ResFinder 4.0 (Zankari et al., 2012), INTEGRALL (Moura et al., 2009), IntegronFinder Galaxy v1.5.1 (Cury et al., 2016)^6^ and ISfinder (Siguier et al., 2006)^7^ programs were utilized. Easyfig 2.2.3 was used for comparative genome alignments and generation of physical maps.

### Conjugation Experiment

Conjugation was performed using the 20 IncC-containing isolates identified in this study as the donor strain respectively and rifampin-resistant *E. coli* EC600 as the recipient strain with selection on nutrient agar plates containing 20 mg/L rifampin and 8 mg/L florfenicol. Positive transconjugants were characterized by assessing mobilization of *repA* gene by PCR with the C-F/R primer pairs described above and determination of the antimicrobial resistance profile.

### Nucleotide Sequence Accession Numbers

The nucleotide sequences of 20 IncC plasmids and isolates in this study were submitted to GenBank and the assigned accession numbers are listed in Table 1 and Supplementary Table S1, respectively.

**TABLE 1:** IncC plasmids characterized in this study.

Type	Plasmid	Accession number	orf1832/orf1847	*rhs1*/*rhs2*	i1*^*a*^*	i2	Organism	Total length (bp)	Length of backbone (bp)	Mean G + C content (%)	Total number of ORFs
Type 1/2a	pEC1-1/2a	MT551208	orf1832	*rhs1*	NP	i2	*E. coli*	139,489	113,808	52.1%	184
Type 1/2a	pEC2-1/2a	MT559985	orf1832	*rhs1*	NP	i2	*E. coli*	247,388	117,312	52.2%	314
Type 1/2a	pEC3-1/2a	MT559986	orf1832	*rhs1*	NP	i2	*E. coli*	160,789	117,312	52.6%	204
Type 1/2a	pEC5-1/2a	MT559988	orf1832	*rhs1*	NP	i2	*E. coli*	146,625	112,022	52.4%	191
Type 1/2a	pEC6-1/2a	MT559989	orf1832	*rhs1*	NP	i2	*E. coli*	214,513	117,312	52.5%	302
Type 1/2a	pEC7-1/2a	MT559990	Δorf1832	Δ*rhs1*	NP	i2	*E. coli*	93,442	74,054	52.0%	129
Type 1/2a	pEC8-1/2a	MT559991	orf1832	*rhs1*	NP	i2	*E. coli*	157,847	117,312	52.2%	204
Type 1/2a	pEC10-1/2a	MT559993	Δorf1832	*rhs1*	NP	i2	*E. coli*	110,589	74,541	52.7%	153
Type 1/2a	pEC13-1/2a	MT559996	Δorf1832	Δ*rhs1*	NP	i2	*E. coli*	88,958	70,058	51.8%	128
Type 1/2a	pKC1-1/2a	MT559999	orf1832	*rhs1*	NP	i2	*K. pneumoniae*	116,633	79,954	52.6%	153
Type 1/2a	pKC2-1/2a	MT560000	orf1832	*rhs1*	NP	i2	*K. pneumoniae*	124,795	79,962	52.6%	156
Type 1/2a	pSC1-1/2a	MT560003	orf1832	*rhs1*	NP	i2	*S. enterica*	147,359	88,201	53.5%	189
Type 1/2a	pPC1-1/2a	MT560002	orf1832	*rhs1*	NP	i2	*P. mirabilis*	153,373	117,312	52.3%	197
Type 1/2b	pKC3-1/2b	MT560001	Δorf1832	*rhs1*	+	–	*K. pneumoniae*	193,802	123,809	52.6%	252
Type 1/2b	pCC1-1/2b	MT559998	–	*rhs1*	+	–	*C. braakii*	180,389	110,009	52.6%	242
Type 1b	pEC4-1b	MT559987	orf1832	*rhs1*	–	–	*E. coli*	89,953	62,552	52.1%	124
Type 1b	pEC11-1b	MT559994	orf1832	*rhs1*	–	–	*E. coli*	166,437	116,870	52.5%	217
Type 1b	pEC12-1b	MT559995	–	*rhs1*	–	–	*E. coli*	69,554	50,286	53.1%	100
Type 1b	pEC14-1b	MT559997	orf1832	*rhs1*	–	–	*E. coli*	167,577	70,058	52.6%	220
Type 2	pEC9-2	MT559992	orf1847	*rhs2*	i1	i2	*E. coli*	137,087	97,100	52.9%	186

*^a^NP = region surrounding i1 location was not present.*

## Results and Discussion

### The Strains Carrying IncC

**FIGURE 1:**
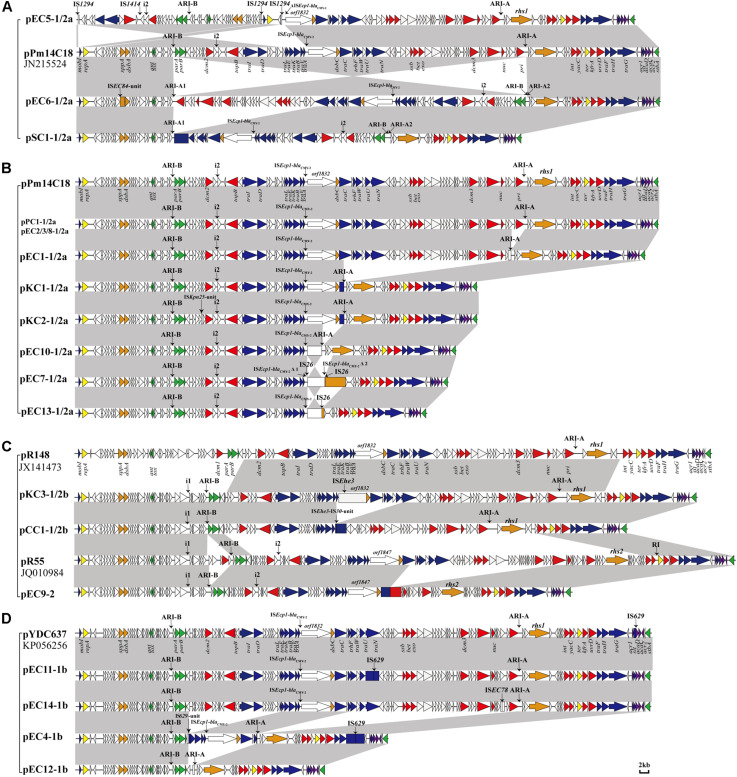
Comparison of linear maps of the IncC plasmids. **(A)** The large sequence inversions mediated by IS-mediated found in the 3 type 1/2a IncC plasmids. **(B)** The remaining type 1/2a IncC plasmids. **(C)** The type 1/2b and type 2 IncC plasmids. **(D)** The type 1b plasmids. The GenBank accession numbers of the reference type 1a pR148, type 1b pYDC637, type1/2a pPm14C18 and type 2 reference pR55 are JX141473, KP056256, KU605240 and JQ010984. Genes and ORFs are shown as arrows, and their orientations of transcription are indicated by the arrowheads. The truncated genes are shown as rectangle. The positions of accessory modules, and the i1 or i2 insertions are indicated by vertical arrows. Genes coding for proteins are colored according to the following key: blue, conjugative transfer; yellow, replication; green, plasmid maintenance; red, DNA metabolism; purple, regulation; orange, other functional genes; white, no known function. Shared regions with above 99.9% identity are indicated by shading.

A total of 20 strains (20/870 total, 2.3%) were determined for carrying IncC plasmids. The positive strains included 14 *E. coli* strains (14/369 total, 3.8%), 3 *K. pneumoniae* strains (3/212 total, 1.4%), 1 *S. enterica* strain (1/125 total, 0.8%), 1 *P. mirabilis* strain (1/56 total, 1.8%), and 1 *C. braakii* strain (1/38 total, 2.6%). 14 *E. coli* isolates were identified as 13 different sequence types (STs) in this study, and on minimum, 11 and maximum, 49,317 SNPs were identified when compared with each other. The two ST4663 *E. coli* strains (EC2 and EC8) were isolated from the same poultry farms in different years, and there were 11 SNPs between them, revealing that they were almost identical. Similarly, the two ST423 *K. pneumoniae* isolates (KC1 and KC2) seemed to spread by clonal expansion (8 SNPs) in another poultry farms. As shown in Figures 1, 3, due to the insertion of IS-mediated unit in backbone or resistance islands (the IS*26*-unit in ARI-B of pEC2-1/2b, and the IS*Kpn25*-unit in backbone of pKC2-1/2a), the IncC plasmids carried by the clonal isolates are structurally different. The minimal inhibitory concentrations (MICs) of the individual strain were listed in Supplementary Table S1, and all the 20 isolates were MDR (defined as resistant to three or more classes of antibiotics).

### Sequence Overview of IncC

The 20 plasmids varied in size from 69.6 to 247.4 kb, with G + C content that ranged from 51.8% to 53.5%, and 100 to 314 ORFs predicted by RAST. Based on four key differences of the IncC backbone (i1, i2, R1 and R2), 15 of the identified IncC plasmids were recognized as type 1/2 hybrid (Table 1). Based on the presence of i1 or i2, the plasmids were further divided into different subtypes (see section “Backbone Features of IncC”). The remaining four IncC plasmids were assigned as type 1, and one was identified as a type 2 IncC plasmid (Table 1). Using the BLAST program, the plasmid sequences were analyzed together with the reference type 1a pR148, type 1b pYDC637 (Lee et al., 2015), type1/2a pPm14C18 (Lei et al., 2017), and type 2 pR55. The pairwise comparison of backbone sequences showed that these 24 plasmids displayed >99% nucleotide identity with ≥42% coverage among the same plasmid type, and had >97% nucleotide identity with ≥38% coverage between different plasmid types (Supplementary Table S3). The 20 plasmids possessed the relatively conserved backbone organization, corresponding to mobilization, replication, maintenance, metabolism, regulation, and some other functional genes. It is in agreement with the findings of previous studies (Fricke et al., 2009), indicating that the core backbones of IncC plasmids are highly syntenic. However, the insertion of accessory modules always led to loss or disruption of functional genes (show in the Figure 1 and Supplementary Table S4). All 20 plasmids identified in this study contained accessory modules, with collections of resistance genes, but the insertions with different profiles ranging from the simple to the very complex (see section “Functional Genes of IncC Plasmids”).

### Backbone Features of IncC

In this study, 15 IncC plasmids were identified as type 1/2 hybrid. Thirteen of these plasmids (Figures 1A,B) contain the backbone features similar to the backbone of plasmid pPm14C18 (Lei et al., 2017), so were designated type 1/2a plasmids. These plasmids harbor the type 2 version of the additional sequence i2 and the type 1 versions of R1 (*orf1832*) and R2 (*rhs1*). In four type 1/2a plasmids, pEC5-1/2a, pEC6-1/2a, pEC7-1/2a and pSC1-1/2a, IS-mediated (IS*26* or IS*1294*) recombination resulted in different sequence inversions of the genome, which were similar to the inversion event in previously described study (such as those in p427113-Ct1/2 and pA1763-Ct2) (Cheng et al., 2019). But more complicated, in pEC6-1/2a and pSC1-1/2a, there were the larger-scale IncC genome inversion mediated by IS*26* between the ARI-B and ARI-A. The large genetic fragments of the backbone region with accessory modules (including multiple inversions arising from within ARI-A, ARI-B and the IS*Ecp1-bla*_*CMY–*__2_ island) were positioned in inverse orientation relative to the other plasmids in this group (Figure 1A). Another subtype was comprised of plasmids pKC3-1/2b and pCC1-1/2b, type 1/2b, which contained *orf1832*, *rhs1*, and additional type 2 sequence i1 (Figure 1C). Analysis of the genome of pKC3-1/2b (GenBank accession number MT560001) by Blast analysis revealed that the backbone sequence from 0-bp to 50,800-bp and from 175,375-bp to 193,802-bp showed 99.97% nucleotide identity to the corresponding backbone region of type 2 IncC plasmid pR55 (GenBank accession no. JQ010984). The remaining backbone, from 50,801-bp to 175,374-bp, showed 99.99% nucleotide identity to the corresponding backbone region of type 1 IncC plasmid pR148 (GenBank accession no. JX141473). There were different backbone features in the 15 type 1/2 hybrid IncC plasmids, suggesting various homologous recombination between type 1 and type 2 IncC, with IS-mediated rearrangements increasing the diversity of the IncC plamids.

Like type 1b IncC plasmid pYDC637 (GenBank accession no. KP056256), the four type 1b IncC plasmids identified in this study (pEC4-1b, pEC11-1b, pEC12-1b, pEC14-1b) (Figure 1D) lacked the SNPs, which are typically concentrated in a 14.5-kb part of the type 1a IncC genome (Ambrose et al., 2018b). In addition, compared to pR55, the only type 2 IncC plasmid, pEC9-2, contained an extremely simple backbone due to the deletion of a large section of the backbone regions, but still retained all the characteristics of type 2 plasmids in the sequences of i1, i2, *orf1847*, and *rhs2*. This plasmid had a deletion of up to 27.6-kb between the *traC* and *nuc* genes relative to pR55, but no related mobile elements were detected (Figure 1C).

Structures of ARI-B and ARI-A are sometimes associated with backbone deletion (Table 2), and this process is mostly mediated by IS*26*. Of the IncC plasmids identified here, the most commonly seen event was a 10,984-bp IS*26*-mediated deletion arising within the ARI-B, which was in agreement with previously described ARI-B (Harmer and Hall, 2015). This deletion was identified in all 13 of the type 1/2a and all four of the type 1b plasmids identified here, but the configuration of ARI-B was slightly different in some plasmids (Table 2). Another event was a 4,477 bp backbone deletion upstream from the *parA* gene, found in the two type 1/2b plasmids, pKC3-1/2b and pCC1-1/2b, and the one type 2 plasmid, pEC9-2, which was associated with the ARI-B forms containing the IncN-related fragment previously characterized (Harmer and Hall, 2015; Figures 3E,F). Similarly, IS*26*-mediated backbone deletions with various sizes can often be found at the upstream of the ARI-A (Table 2). Additionally, other acquired regions (IS elements or transposons) were identified that interrupt or delete the backbone of IncC genome (Table 2).

**TABLE 2:** Accessory modules in IncC plasmids.

Plasmid	ARI-A		ARI-B		IS*Ecp1-bla*_*CMY–*__2_	Others	
	Resistance	Deletion(bp)	Variant	Deletion (bp)		IS	Deletion(bp)
pEC1-1/2a	pDU_*mer*_	3,499	A	10,984	+	–	–
pEC2-1/2a	*tmrB*, *aac(3)-IIa*, *erm*(B), *mph*(A)	–	D	10,984	interrupted	IS*1*	–
pEC3-1/2a	*aacA7, sul1, Inu(F), aadA22*, pDU_*mer*_	–	A	10,984	+	–	–
pEC5-1/2a	pDU_*mer*_	–	A	10,984*^*b*^*	interrupted	IS*1294*, IS*1414*	5,303
pEC6-1/2a	*sul3*, *aadA2*, *dfrA12*, *bla*_*TEM–*__1__*B*_, *mph*(A), ΔTn*21*_*mer*_	–	C	10,984*^*c*^*	+	IS*Ec84*-unit	–
pEC7-1/2a	–	–	A	10,984	interrupted	IS*26*	43,217
pEC8-1/2a	*tmrB*, *aac(3)-IIa*, *erm*(B), *mph*(A)	–	A	10,984	interrupted	IS*1*	–
pEC10-1/2a	*erm*(B), *mph*(A), pDU_*mer*_	41,765	A	10,984	+	–	–
pEC13-1/2a	–	–	B	10,984	+	IS*26*	47,248
pKC1-1/2a	*aadB*, *cmlA1*, *bla*_*TEM–*__1__*B*_, ΔTn*21*_*mer*,_ ΔpDU_*mer*_	37,361	A	10,984	+	–	–
pKC2-1/2a	*aadB*, *cmlA1*, *bla*_*TEM–*__1__*B*_, ΔTn*21*_*mer*,_ ΔpDU_*mer*_	37,361	A	10,984	+	IS*Kpn25*-unit	–
pPC1-1/2a	*aacA7*, *sul1*, ΔpDU_*mer*_	–	A	10,984	+	–	–
pSC1-1/2a	*mph*(A), *qepA*, *dfrA12*, *aadA2, sul1, qnrS1, bla*_*TEM–*__1__*B*_, ΔTn*21*_*mer*,_ pDU_*mer*_	29,106	C	10,984*^*c*^*	+	–	–
pKC3-1/2b	*dfrA14*, *arr-2*, *cmlA1, bla*_*OXA–*__10_, *aadA1*, *sul1*, *qnrB4*, *bla*_*DHA–*__1_, *mph*(A), Tn*21*_*mer*_	–	F	4,477	–	IS*3*	–
pCC1-1/2b	*dfrA14*, *arr-2*, *cmlA1, bla*_*OXA–*__10_, *aadA1*, *sul1*, *qnrB4*, *bla*_*DHA–*__1_, *mph*(A), Tn*21*_*mer*_	–	F	4.477	–	IS*3*, IS30	13,790
pEC4-1b	–*^*a*^*	34,760	A	10,984	+	IS*629-unit*	19,544
pEC11-1b	*qnrVC4*, *aac(6’)Ib-cr*, *cmlA1*,*aadA1*, *bla*_*OXA–*__10_, *aadA1*, *dfrA14*, *mph*(A), *aph(3’)-Ia*, pDU_*mer*_	–	A	10,984	+	IS*3*	–
pEC12-1b	*mph*(A)	66,835	A	10,984	–	–	–
pEC14-1b	*qnrVC4*, *aac(6’)Ib-cr*, *cmlA1*,*aadA1*, *bla*_*OXA–*__10_, *aadA1*, *dfrA14*, *mph*(A), *aph(3’)-Ia*, pDU_*mer*_	–	A	10,984	+	IS*66*	–
pEC9-2	–	–	E	4,477	–	–	27,636

*^a^Due to the IS26- mediated recombination, in ARI-A, only ΔIS4321 was retained. ^b^The ARI-B was reversed with the adjacent backbone region. ^c^The IS26 transposition event occurred between the ARI-B and ARI-A.*

### Functional Genes of IncC Plasmids

As shown in Figure 1, the insertion mediated by accessory modules always resulted in the loss or disruption of the functional genes (some *tra* genes and metabolism genes) in varying degrees. The mobilization modules contained type IV secretion system (T4SS, *traLEKBVACWUFHG*) genes (Guglielmini et al., 2013), other *tra* genes (*traIDN* and *trhF*), *mobI* and *slt* genes, and only 8 IncC plasmids (pEC1-1/2a, pEC2-1/2a, pEC3-1/2a, pEC6-1/2a, pEC8-1/2a, pPC1-1/2a, pKC3-1/2b, and pEC14-1b) of this study maintained all these genes intact, but only 7 plasmids the ability to transfer (see section “Transferability and Antimicrobial Susceptibility”). In this study, the genes required for IncC plasmid replication (*repA* and *ter*) and maintenance (*ant*, *tox*, *parAB*, *sta*, and a putative gene, *053*) of all 20 plasmids were complete and uninterrupted at their genome. The *repA* gene, a toxin-antitoxin (TA) system (*ant* and *tox*) and a set of 3 partitioning-related genes (*parA*, *parB* and *053*) have been identified roles for the stability (Hancock et al., 2017), so the integrity of these genes is critical for plasmid maintenance in host.

### Resistance Islands of IncC Plasmids

**FIGURE 2:**
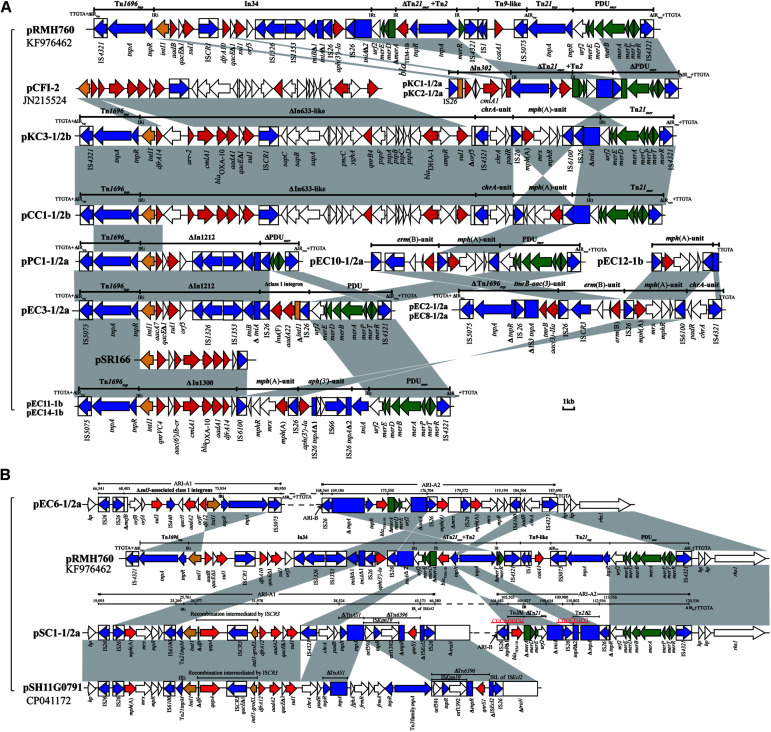
Comparison of ARI-A containing resistance genes. **(A)** The common ARI-A forms. **(B)** The ARI-A forms inverted by IS*26*. The GenBank accession numbers of pRMH760, pCFI-2 (Yim et al., 2013), pSR166, and pSH11G0791 for reference are KF976462, JN215524, KU886277, and CP041172, respectively. Genes and ORFs are shown as arrows, and their orientations of transcription are indicated by the arrowheads. The truncated genes are shown as rectangle, which are indicated with a Δ. The IS elements are indicated by boxes around the blue arrows. Genes coding for proteins are colored according to the following key: blue, transposase genes; yellow, integrase genes; red, antimicrobial resistance genes; green, mercury resistance genes; white, other genes. Shared regions with above 99.9% identity are indicated by shading.

Except for the type 2 plasmid, pEC9-2, and the two type 1/2a plasmids, pEC7-1/2a and pEC13-1/2a, ARI-A islands were identified in the remaining 17 plasmids (Table 2 and Figure 2). The structure of ARI-A in the IncC plasmid pRMH760 (GenBank accession no. KF976462) was the first to be described in detail (Partridge and Hall, 2003; Harmer and Hall, 2017). Similar to pRMH760, there were 10 plasmids in this study have both ends of ARI-A intact, with this sequence flanked by a 5-bp duplication (TTGTA) of the target (Figure 2A, the ARI-A of pEC5-1/2a is not shown). The 10 ARI-A islands were all identified as a complex mosaic structure derived from Tn*1696* and composed of a different class 1 integron and multiple resistance units or transposons (Figure 2), of which the two outermost inverted repeats are interrupted by either IS*4321* or IS*5075* elements. In agreement with the conclusions of a previous study about this form of ARI-A (Harmer and Hall, 2015), these islands include varying resistance island (RI) sequences, and this interruption of the IR effectively “locks” this island in place, making all insertions had the same boundaries in the plasmid backbone and evolution *in situ*. As a result of IS*26*-mediated recombination, the other 7 plasmids contained deletions originating within the island, which removed part of ARI-A and different portions of the backbone adjacent to the upstream of ARI-A (Table 2), resulting in a relatively ARI-A structure. It is also notable that, in pEC6-1/2 and pSC1-1/2, the IS*26* transposition event occurred between ARI-B and ARI-A, resulting in the interruption of ARI-A into two parts (ARI-A1 and ARI-A2), and an inversion of the adjacent genetic fragments of the backbone, with all of ARI-B and parts of ARI-A (ARI-A1) oriented in the inverse orientation (Figures 1A,B). The lack of 8-bp target inverted repeats (IRs) flanking the inversion, suggests that this complex reorganization was mediated by IS*26*. Also due to IS*26*-mediated rearrangements, compared to pRMH760, part of Tn*21*_*mer*_ and the Tn*2* region in ARI-A2 of pSC1-1/2a and pEC6-1/2a (corresponding to bases 105,503-109,980 in accession no. MT560003, and bases 169,184-176,704 in accession no. MT559989) were in inverse orientation, and the inverted part of pSC1-1/2a was flanked by 8-bp IRs (CGCAGGCG).

**FIGURE 3:**
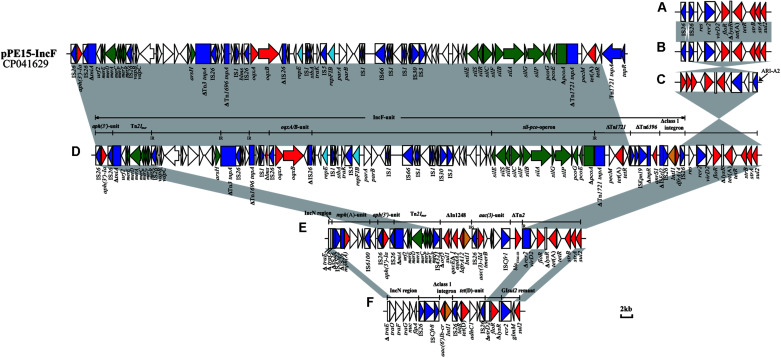
Configurations of ARI-B in IncC. **(A)** The most common ARI-B configuration in this study. **(B)** Configuration present in pEC13-1/2a.**(C)** Configuration present in pEC6-1/2a and pSC1-1/2a. **(D)** Configuration present in pEC2-1/2a. **(E)** Configuration present in pEC9-2. **(F)** Configuration present in pKC3-1/2b and pCC1-1/2b. The GenBank accession number of pPE15 is CP041629. Genes and ORFs are shown as arrows, and their orientations of transcription are indicated by the arrowheads. The truncated genes are shown as rectangle, which are indicated with a Δ. The IS elements are indicated by boxes around the blue arrows. Genes coding for proteins are colored according to the following key: blue, transposase genes; yellow, integrase genes; red, antimicrobial resistance genes; green, mercury resistance genes; light blue, *rep* genes; white, other genes. Shared regions with above 99.9% identity are indicated by shading.

In all 20 IncC plasmids, ARI-B was located upstream of the *parA* gene, and all contained the *sul2*-carrying remnants of GI*sul2* (Nigro and Hall, 2011; Harmer et al., 2017). Except for pKC3-1/2b and pCC1-1/2b, the 18 other plasmids all contain another four resistance genes (*floR*, *tet*(A), *strA*, and *strB*). Thirteen of the plasmids (Table 2 and Figure 3A) contain the most commonly seen configuration of ARI-B (Fig. 7A in the review by Harmer and Hall, 2015), which is associated with a 10,984 bp, IS*26*-mediated deletion of the backbone. The ARI-B of pEC13-1/2 (Figure 3B) was different from variant A (Figure 3A) only by inversion of the short segment between the two IS*26*. In three plasmids with a large inversion (pEC5-1/2a, pEC6-1/2a, and pSC1-1/2a), their ARI-B sequence and the adjacent backbone region were reversed, and the right hand of ARI-B in pEC6-1/2a and pSC1-1/2a was linked to ARI-A2 by one IS*26* (Figures 2A, 3C). There was also a deletion event that removed 4,477 bp of the backbone in pKC3-1/2b, pCC1-1/2b, and pEC9-2, generating a junction between the backbone and a segment derived from the *tra* region of an IncN plasmid, and these plasmids have a dramatically different set of resistance genes, as shown in Figures 3E,F. In pEC2-1/2a (Figure 3D), a distinct ARI-B form contains a 90.0-kb segment surrounded by two IS*26* elements sharing 99.99% nucleotide identity with the IncF plasmid pPE15 (GenBank accession no. CP041629), explaining acquisition of newer metal (*sil-pco*-operon and Tn*21*_*mer*_) and antibiotic (*aph(3’)-Ia*, *oqxA/B*, *tet*(A), *qnrS1*, *dfrA14*) resistance modules with a highly mosaic nature, and suggesting that IS*26* promotes diversity of ARI-B in IncC genome.

In 16 plasmids of this study, as previously reported, the *bla*_*CMY–*__2_ gene was associated with the mobile element IS*Ecp1* in an island that was positioned downstream of the *traA* gene. In four plasmids, the IS*Ecp1* sequence was interrupted by IS*1* or IS*91*. Additionally, pEC7-1/2a contained an inversion of the IS*Ecp1-bla*_*CMY–*__2_ island and *orf1832* mediated by two IS*26* elements in opposite orientation (Figure 1B), resulting in a 43.2-kb deletion of the backbone.

### Transferability and Antimicrobial Susceptibility

We successfully obtained seven transconjugants harboring IncC, indicating successful transfer from wild-type isolates into EC600 through conjugation, as listed in Supplementary Table S5. The antimicrobial susceptibility testing of the transconjugants showed that the *bla*_*CMY–*__2_ gene could be successfully transferred to EC600, and the antimicrobial resistance profiles of the resulting strains are shown in Supplementary Table S5. The seven IncC plasmids are all have an intact mobilization module, but the plasmid pEC6-1/2a, which failed to transfer, carried the unbroken but inverted mobilization modules (tra*IDLEKBVACWUN* and *trhF*). The failure to transfer in the remaining IncC plasmids may reflect the deletion or inversion of the related transfer genes, and the underlying mechanism will be explored in future studies.

## Conclusion

In this study, we characterized 20 IncC plasmids identified from livestock farms in China and determined the antibiotic resistance. The accessory modules insertions always resulted in the loss or disruption of the functional genes (some *tra* genes and metabolism genes) to varying degrees, which might account for conjugation failure of some IncC plasmids. Meanwhile, the evolution of resistance islands via loss and gain of diverse modules within the large resistance regions of ARI-A and ARI-B has driven leading to different resistance phenotypes. Variation existed in the 15 type 1/2 hybrids suggests various homologous recombination between type 1 and type 2 IncC plasmids. In addition to insertion of accessory modules and deletion of backbone regions, IS-mediated rearrangements resulted in large sequence inversions of the MDR regions extending outside the IncC backbone. The identification of the four IncC plasmids with IS-mediated inversion event suggested that IS-mediated rearrangements increase the diversity of IncC genome, especially the IS*26*, and we will pay attention to its role in promoting diversity in future studies.

## Data Availability Statement

The datasets presented in this study can be found in online repositories. The names of the repository/repositories and accession number(s) can be found below: https://www.ncbi. nlm.nih.gov/genbank/, MT551208; https://www.ncbi.nlm.nih. gov/genbank/, MT559985; https://www.ncbi.nlm.nih.gov/gen bank/, MT559986; https://www.ncbi.nlm.nih.gov/genbank/, MT559988; https://www.ncbi.nlm.nih.gov/genbank/, MT559989; https://www.ncbi.nlm.nih.gov/genbank/, MT559990; https://www.ncbi.nlm.nih.gov/genbank/, MT559991; https://www.ncbi. nlm.nih.gov/genbank/, MT559993; https://www.ncbi.nlm.nih. gov/genbank/, MT559996; https://www.ncbi.nlm.nih.gov/gen bank/, MT559999; https://www.ncbi.nlm.nih.gov/genbank/, MT560000; https://www.ncbi.nlm.nih.gov/genbank/, MT560003; https://www.ncbi.nlm.nih.gov/genbank/, MT560002; https://www.ncbi.nlm.nih.gov/genbank/, MT560001; https://www.ncbi. nlm.nih.gov/genbank/, MT559998; https://www.ncbi.nlm.nih. gov/genbank/, MT559987; https://www.ncbi.nlm.nih.gov/gen bank/, MT559994; https://www.ncbi.nlm.nih.gov/genbank/, MT559995; https://www.ncbi.nlm.nih.gov/genbank/, MT559997; https://www.ncbi.nlm.nih.gov/genbank/, MT559992; https://www.ncbi.nlm.nih.gov/genbank/, JACXRF000000000; https://www.ncbi.nlm.nih.gov/genbank/, JACXRG000000000; https://www.ncbi.nlm.nih.gov/genbank/, JACXRH000000000; https://www.ncbi.nlm.nih.gov/genbank/, JACXRI000000000; https://www.ncbi.nlm.nih.gov/genbank/, JACXRJ000000000; https://www.ncbi.nlm.nih.gov/genbank/, JACXRK000000000; https://www.ncbi.nlm.nih.gov/genbank/, JACXRL000000000; https://www.ncbi.nlm.nih.gov/genbank/, JACXRM000000000; https://www.ncbi.nlm.nih.gov/genbank/, JACXRN000000000; https://www.ncbi.nlm.nih.gov/genbank/, JACXRO000000000; https://www.ncbi.nlm.nih.gov/genbank/, JACXRP000000000; https://www.ncbi.nlm.nih.gov/genbank/, JACXRQ000000000; https://www.ncbi.nlm.nih.gov/genbank/, JACXRR000000000; https://www.ncbi.nlm.nih.gov/genbank/, JACXRS000000000; https://www.ncbi.nlm.nih.gov/genbank/, JACXRT000000000; https://www.ncbi.nlm.nih.gov/genbank/, JACXRU000000000; https://www.ncbi.nlm.nih.gov/genbank/, JACXRV000000000; https://www.ncbi.nlm.nih.gov/genbank/, JACXRW000000000; https://www.ncbi.nlm.nih.gov/genbank/, JACXRE000000000; https://www.ncbi.nlm.nih.gov/genbank/, JACXSK000000000.

## Ethics Statement

This study was carried out in accordance with the recommendation of ethical guidelines of Sichuan University. The protocol was approved by the Sichuan University Animal Ethics Committee. Individual informed consent for the use of samples was obtained from all the animal owners.

## Author Contributions

YZ, C-WL, and XC performed the experiments. YZ, T-GY, J-WY, W-LH, and XM analyzed the data. YZ and C-WL wrote the manuscript. YZ and C-WL conceived of the study. All authors contributed to manuscript revision and approved the final manuscript.

## Conflict of Interest

The authors declare that the research was conducted in the absence of any commercial or financial relationships that could be construed as a potential conflict of interest.

## Footnotes

1 https://www.who.int/news-room/feature-stories/ten-threats-to-global-health-in-2019

2 https://github.com/rrwick/Unicycler

3 https://cge.cbs.dtu.dk/services/MLST/

4 https://cge.cbs.dtu.dk/services/CSIPhylogeny/

5 http://www.ncbi.nlm.nih.gov/BLAST/

6 https://galaxy.pasteur.fr/root?tool_id=toolshed.pasteur.fr%2Frepos%2Fkhillion%2Fintegron_finder%2Fintegron_finder%2F1.5.1

7 https://www-is.biotoul.fr/
