# Supplementary material for: Characterization of IncC Plasmids in Enterobacterales of Food-Producing Animals Originating From China
Source: Front Microbiol. 2020 Oct 27;11:580960. doi: 10.3389/fmicb.2020.580960 (PMC7652850; doi:10.3389/fmicb.2020.580960)
Supplement: Supplementary file 2 [file Table_2.DOCX]

| Sample | Mean Reads Length (bp) | Data size (G) | Genome size (M) | Depth |
| --- | --- | --- | --- | --- |
| EC1 | **11,026** \| (150:150) | **2.32** \| 1.11 | 5.25 | **442×** \| 211**×** |
| EC2 | **6,231** \| (150:150) | **0.96** \| 1.10 | 4.83 | **199×** \| 228**×** |
| EC3 | **9,099.4** \| (150:150) | **1.23** \| 1.25 | 4.99 | **246×** \| 251**×** |
| EC4 | **12,420** \| (150:150) | **1.13** \| 1.39 | 4.92 | **229×** \| 283**×** |
| EC5 | **9,884** \| (150:150) | **2.48** \| 1.28 | 5.29 | **468×** \| 242**×** |
| EC6 | **4947** \| (150:150) | **0.99** \| 1.86 | 5.29 | **187×** \| 352**×** |
| EC7 | **4,561** \| (150:150) | **0.98** \| 2.09 | 4.96 | **197×** \| 421**×** |
| EC8 | **9,431.8** \| (150:150) | **1.43** \| 1.26 | 5.31 | **269×** \| 237**×** |
| EC9 | **10,239** \| (150:150) | **0.98** \| 2.07 | 4.84 | **202×** \| 428**×** |
| EC10 | **5,027** \| (150:150) | **0.98** \| 1.96 | 4.76 | **206×** \| 412**×** |
| EC11 | **11,995** \| (150:150) | **1.00** \| 2.12 | 5.72 | **175×** \| 371**×** |
| EC12 | **13,439** \| (150:150) | **0.95** \| 1.62 | 4.93 | **193×** \| 329**×** |
| EC13 | **13,439** \| (150:150) | **1.01**\| 1.88 | 4.92 | **206×** \| 382**×** |
| EC14 | **7,970.6** \| (150:150) | **1.82** \| 1.77 | 4.95 | **368×** \| 358**×** |
| KC1 | **22,771** \| (150:150) | 1.14 \| 2.24 | 5.59 | **204×** \| 401**×** |
| KC2 | **10,074** \| (150:150) | **1.16** \| 2.32 | 5.37 | **216×** \| 432**×** |
| KC3 | **9,721.5** \| (150:150) | **1.08** \| 2.27 | 5.68 | **190×** \| 400**×** |
| SC1 | **18,595** \| (150:150) | **0.89** \| 2.32 | 4.93 | **181×** \| 471**×** |
| PC1 | **9,499.3** \| (150:150) | **1.87** \| 1.59 | 4.12 | **454×** \| 386**×** |
| CC1 | **4,941** \| (150:150) | **0.94** \| 2.04 | 5.17 | **181×** \| 395**×** |

**Table S2** Summary statistics for reads generated by Nanopore (numbers in bold) and Illumina assemblies.
