# Supplementary material for: Characterization of IncC Plasmids in Enterobacterales of Food-Producing Animals Originating From China
Source: Front Microbiol. 2020 Oct 27;11:580960. doi: 10.3389/fmicb.2020.580960 (PMC7652850; doi:10.3389/fmicb.2020.580960)
Supplement: Supplementary file 5 [file Table_5.DOCX]

| Transconjugant | MIC(μg/mL) | | | | | | | | | | | | | |
| --- | --- | --- | --- | --- | --- | --- | --- | --- | --- | --- | --- | --- | --- | --- |
|  | AMP | FOX | CTX | CRO | IPM | AMC | FFC | CIP | GEN | DOX | SUL | TMP | FOS | POL |
| EPC1 | ≥256 | 32 | 2 | 2 | ≤1 | 16/8 | ≥128 | ≤1 | ≤1 | ≥128 | ≥512 | 4 | ≤16 | ≤1 |
| EEC1 | ≥256 | 32 | ≤1 | 2 | ≤1 | 16/8 | ≥128 | ≤1 | ≤1 | ≥128 | ≥512 | 2 | ≤16 | ≤1 |
| EEC2 | ≥256 | 32 | 2 | ≤1 | ≤1 | 32/16 | ≥128 | 4 | 16 | ≥128 | ≥512 | ≥16 | ≤16 | ≤1 |
| EEC3 | ≥256 | 16 | ≤1 | ≤1 | ≤1 | 16/8 | ≥128 | ≤1 | ≤1 | ≥128 | ≥512 | 2 | ≤16 | ≤1 |
| EEC8 | ≥256 | 16 | 2 | ≤1 | ≤1 | 16/8 | ≥128 | ≤1 | 16 | ≥128 | ≥512 | 2 | ≤16 | ≤1 |
| EKC3 | ≥256 | 16 | ≤1 | 2 | ≤1 | 32/16 | ≥128 | 4 | 8 | ≥128 | ≥512 | ≥16 | ≤16 | ≤1 |
| EEC14 | ≥256 | 16 | 2 | ≤1 | ≤1 | 32/16 | ≥128 | 4 | 8 | ≥128 | ≥512 | ≥16 | ≤16 | ≤1 |
| EC600 | ≤1 | ≤1 | ≤1 | ≤1 | ≤1 | ≤4/2 | 2 | ≤1 | ≤1 | 2 | ≤16 | ≤2 | ≤16 | ≤1 |

Table S5. Antimicrobial resistance profile of transconjugants
